# Relationships Between Heart Rate Variability, Occupational Performance, and Fitness for Tactical Personnel: A Systematic Review

**DOI:** 10.3389/fpubh.2020.583336

**Published:** 2020-11-09

**Authors:** Colin Tomes, Ben Schram, Robin Orr

**Affiliations:** ^1^Faculty of Health Science and Medicine, Bond University, Robina, QLD, Australia; ^2^Tactical Research Unit, Bond University, Robina, QLD, Australia

**Keywords:** injury prevention and reduction, occupational stress and mental-physical health, physiological monitoring data, military, police, firefighting, first responder, occupational fitness

## Abstract

**Objectives:** Heart Rate Variability has gained substantial interest in both clinical and athletic settings as a measurement tool for quantifying autonomic nervous system activity and psychophysiological stress. However, its uses in tactical work settings, such as military, police, and firefighting environments, remain controversial. Given the physical, mental, and emotional stress public safety personnel face both operationally and in training, heart rate variability measurement may be key in promoting their health, safety and operational effectiveness.

**Methods:** This study identified, critically appraised, and summarized primary studies investigating relationships between heart rate variability and outcomes of interest to tactical personnel. Key literature databases were searched, and quality assessment checklists were applied to analyze retained literature. The results of the screening and assessment processes, along with key data extracted from each study were summarized and tabulated. Research gaps were also identified to facilitate improvements to how tactical personnel and health or performance providers may best utilize heart rate variability to monitor or promote personnel health and performance, and thereby facilitate public safety.

**Results:** Twenty studies were included and were all of generally high quality. Cohort size, length of follow-up, measurement objectives, data acquisition, and data analysis all varied considerably across studies, precluding meta-analysis. However, study results correlating heart rate variability and relevant outcomes indicated that overall, heart rate variability is an effective indicator of key fitness and performance elements in the tactical work setting.

**Conclusions:** Heart rate variability can be an effective health and performance tool in tactical work environments. However, measurement methods must be carefully selected and applied. Further research is required to understand causal relationships. Specifically, larger cohort inclusion and the isolation and study of specific variables unique to public safety work and training may improve the effectiveness of heart rate variability measurement to provide meaningful information to end users and providers.

## Introduction

Heart rate is typically described as the number of beats of the heart per minute, however, the time between beats in a healthy heart is highly variable; the heart is under constantly varying regulation modulating its activity from the two complimentary branches of the autonomic nervous system (ANS): the sympathetic and parasympathetic nervous systems (1, 2). The autonomic nervous system is the efferent branch of the peripheral nervous system responsible for regulating most unconscious bodily activities; respiration, distribution of blood flow, digestion, pupillary response, waste excretion, arousal, and heart rate (1). The ANS is itself composed of two complimentary pathways that typically act antagonistically to maintain homeostasis. The sympathetic nervous system originates from the thoracic and upper lumbar vertebrae, and governs response that require immediate action, such as the “fight or flight” response to acute stress. The parasympathetic nervous system is characterized as a more slow-moving, dampening response, and governs actions that do not typically require immediate action (1).

With respect to the heart specifically, the sympathetic nervous system increases heart rate, which will decrease the variation in time between beats. This is accomplished not only through direct innervation, in which the muscle is stimulated to contract more quickly and more forcefully, but also through catecholamines secreted via the adrenal glands (1). The parasympathetic nervous system regulates the heart through the vagus nerve, slowing conduction of the sinoatrial node and contributing to respiratory sinus arrhythmia, the natural increase in heart during inspiration and decrease during expiration (3).

Disruption of the balance between sympathetic and parasympathetic influences on the cardiovascular system can lead to devastating health consequences (4, 5). These disruptions have been described as excess allostatic load (6). Allostatic loading occurs in situations of either energy insufficiency, in which energy demand exceeds supply, and focus must be diverted wholly to survival and the maintenance of a positive energy balance, or in situations with energy abundance, but social conflict or disruption (6). While the two types of allostatic load differ to some extent, both disrupt cardiovascular autonomic balance, and are associated with the development of chronic diseases, such as excessive inflammation, chronic pain, diabetes, asthma, fatigue, depression, and anxiety (5). Acute injuries, such as concussion, may also contribute to elevated allostatic load (7), and consequent disruptions to cardiac autonomic balance. Conversely, higher levels of physical fitness are associated with a more optimal cardiovascular autonomic balance (8).

HRV has been identified as a valid measurement of autonomic nervous system regulation and has both time and cost advantages over other methods, such as biomarker testing, while also being noninvasive. With recent technological developments, HRV data are now available and interpretable by coaches and individual athletes, not requiring specialist appointments to read or understand results (9). Correlations between HRV measurements and process or outcome measures are allowing for readily accessible insights to valuable health and fitness data (10, 11) and have been associated with the prediction of morbidity from the chronic and acute disorders mentioned above. Individuals at high risk for the development of ANS dysregulation or allostatic loading and consequent morbidity may therefore benefit from HRV monitoring. In the athletic performance setting, HRV data has been proven useful in improving the precision of energy expenditure estimates (12). More recently, HRV has been investigated as a means of quantifying fatigue and recovery levels, detecting overuse injuries, and calibrating training loads (11, 13, 14). Research continues to emerge linking HRV domains to human performance optimization efforts, which have themselves recently gained interest within the tactical community.

Tactical personnel, individuals who have sworn to protect and serve their communities (15), and who may place their own health and safety at risk in execution of those duties, are often faced with a multitude of unique challenges that often result in stresses to their autonomic nervous systems (16, 17), and may benefit from access to the fitness and health data HRV analyses can provide. For example, police officers and other first responders (firefighters, emergency medical responders) may be sedentary for the majority of their time on duty but could be called with little to no warning into situations of extreme danger and physiological stress (18). The ability to monitor physiological response during these events may help protect their health. Likewise, military personnel, while often prepared in advance for deployment, are exposed to high levels of physical, mental, and emotional stress for prolonged periods of time (19), and may thus also benefit from physiological data measurement and analysis. Furthermore, tactical personnel are also regularly subjected to operations in austere environments, dysregulated sleep and poor nutrition (20, 21). As a result of these occupational demands, literature has reported the risk of cardiovascular disease, especially in police officers, may be greater than that of the general population (22). Conversely, many tactical personnel perform at extraordinary levels of physical performance and may seek to optimize their training, avoid overtraining and maximize the balance between training and operational demands (23).

As a result, HRV applications developed in both clinical and athletic settings may be of interest to tactical organizations seeking an inexpensive and noninvasive means of monitoring the health and fitness of their personnel, both in training and in operations. However, the unique requirements of working as a tactical professional may dictate that functional inferences derived from HRV measurements originally developed in athletic or clinical populations may not always generalize to tactical populations (15). To date, no comprehensive, systematic reviews of the literature examining associations between occupational fitness, operator or trainee health, or occupational performance and heart rate variability analyses for tactical personnel have been conducted. Therefore, the aim of this systematic review was to identify primary studies examining relationships between HRV and relevant health and operational outcomes specifically in tactical personnel, critically appraise the methodological quality of the identified studies, and summarize the results to inform tactical professionals and those in the health and performance fields supporting tactical professionals.

## Survey Methodology

### Search Method

**Figure 1:**
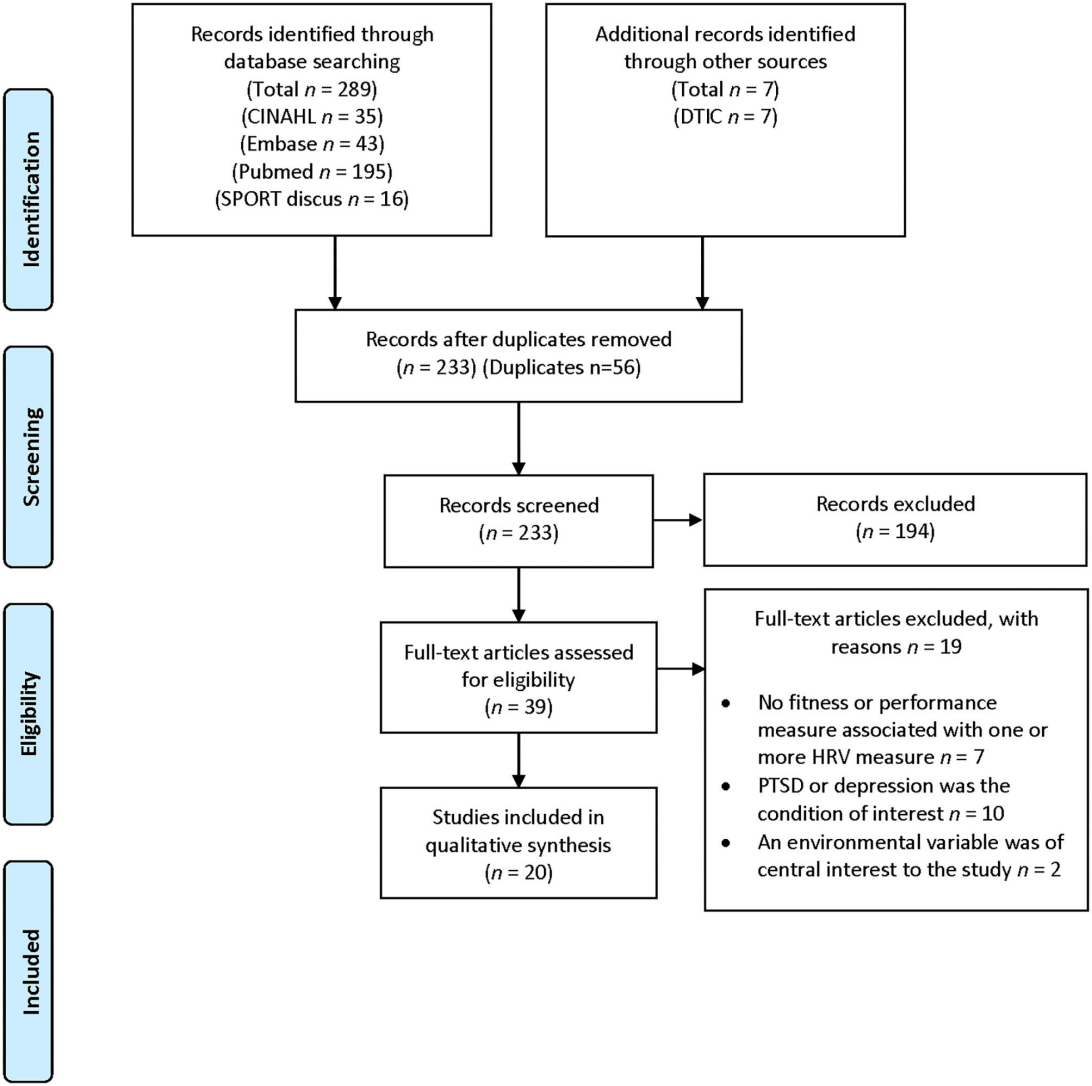
PRISMA flow diagram.

Prior to conducting the initial search, this study was registered with PROSPERO (ID: 153293). The PRISMA guidelines for systematic reviews were followed and the outcome of each step can be found in Figure 1 (24). A rapid search using a set of predetermined keywords was conducted to determine relevant subject heading terms and develop a sample of salient articles to guide a detailed search strategy.

The PICO strategy developed to construct the organization and syntax of search terms was as follows: Patient(s); tactical personnel, and derivative, related, or more specific terms that would capture research conducted with public safety operators or trainees, Intervention; while no interventional studies were expected, given the observational nature of HRV analysis and the research aim, if studies compared HRV to another health or performance monitoring modality, results could be considered provided details of the HRV analysis were included. The control/comparison was not limited or specified; any comparison method could be included, provided the specific details of HRV monitoring were reported. For outcomes, a wide variety of health and occupational outcomes were considered, including physical fitness/injury, mental/emotional stress, or recovery, resilience or job performance.

Each database (Pubmed, CINAHL, Embase, and SportDiscus) was then sequentially searched by a single author (CT) using database heading terms, Boolean operators and available filters. A second author independently verified the search results (RO). The Defense Technical Information Center (DTIC) was also searched to capture gray literature not indexed in traditional academic databases. The DTIC was utilized as an expedient means of capturing information relevant to the research question and population of interest that was not peer-reviewed but was still likely to contribute to the aim of the study. The final search was executed and verified on 9 October 2019. Details of the finalized search strategy and initial results can be seen in Table 1. Duplicates were screened using EndNote software (Clarivate Analytics, Philadelphia, PA, USA), while additional duplicates were removed manually.

**Table 1:** Search strategy and initial results.

**Database**	**Population**	**Target variable**	**Outcome**	**Results**
Pubmed	((“Military Personnel” [Mesh] OR “Emergency Responders”[Mesh] OR “Police” [All Fields] OR “firefighters” [All Fields] OR “Sheriff” [All Fields] OR “Patrol Officer” [All Fields] OR “Law Enforcement”[All Fields] OR “tactical athlete” [All Fields] OR “cadet” [All Fields] OR “agent”[All Fields] OR “recruit” [All Fields] OR “FBI” [All Fields] OR “officer” [All Fields]) OR defense OR defense	(“HRV”[All Fields] OR “Heart Rate Variability” [All Fields] OR “Heart Rate Interval” [All Fields] OR “RR variability” [All Fields] OR “cycle length variability” [All Fields] OR “heart period variability” [All Fields] OR “autonomic function” [All Fields] OR “vagal control” [All Fields]))	Exercise “[All Fields]” OR Physical Exertion “[All Fields] OR Physical Fitness “[All Fields]” OR BMI “[All Fields]” OR Body Mass Index “[All Fields]” OR Body Constitution “[All Fields]” OR Stress “[All Fields]” OR Work Performance “[All Fields]” OR endurance [All Fields] OR fitness [All Fields] OR strength[All Fields] OR (“cumulative trauma disorders” [MeSH Terms] OR (“cumulative” [All Fields] AND “trauma” [All Fields] AND “disorders” [All Fields]) OR “cumulative trauma disorders” [All Fields] OR (“overuse” [All Fields] AND “injury” [All Fields]) OR “overuse injury” [All Fields]) OR ((“Stress” [Journal] OR “stress” [All Fields]) AND (“wounds and injuries” [MeSH Terms] OR (“wounds” [All Fields] AND “injuries” [All Fields]) OR “wounds and injuries” [All Fields] OR “injury” [All Fields])) OR recovery [All Fields] OR (“fatigue” [MeSH Terms] OR “fatigue” [All Fields]) OR readiness [All Fields] OR resilience[All Fields] OR overtraining[All Fields] OR hardiness [All Fields] OR exhaustion[All Fields])	195
CINAHL	(MH “Military Personnel”) OR (MH “Emergency Medical Technicians”) OR (MH “Police”) OR (MH “Firefighters”) OR “Military Personnel” OR Police OR “Emergency Responder” OR “Firefighter” “Sheriff” OR “Patrol Officer” OR “Law Enforcement” OR “tactical athlete” OR “cadet” OR “agent” OR “recruit” OR “FBI” OR “officer” OR defense OR defense	“HRV” OR (MH “Heart Rate Variability”) OR “Heart Rate Interval” OR “RR variability” OR “cycle length variability” OR “heart period variability” OR “autonomic function” OR “vagal control”	(MH “Exercise”) OR (MH “Body Mass Index”) OR (MH “Hardiness”) OR (MH “Athletic Performance”) OR (MH “Job Performance”) OR (MH “Physical Performance”) OR (MH “Physical Fitness”) OR (MH “Fatigue”) OR (MH “Mental Fatigue”) OR (MH “Muscle Fatigue”) OR (MH “Recovery”) OR (MH “Recovery, Exercise”) OR (MH “Stress”) OR (MH “Stress Disorders, Post-traumatic”) OR (MH “Stress, Occupational”) OR (MH “Stress, Physiological”) OR (MH “Fractures, Stress”) OR (MH “Stress, Psychological”) OR (MH “Overtraining Syndrome”) OR strength OR endurance OR resilience OR overuse OR “work performance” OR readiness OR exhaustion	35
SPORTDiscus	“Military Personnel” OR “Emergency Responders” OR “Police” OR “firefighters” OR “Sheriff” OR “Patrol Officer” OR “Law Enforcement” OR “tactical athlete” OR “cadet” OR “agent” OR “recruit” OR “FBI” OR “officer” OR defense OR defense	“HRV” OR “Heart Rate Variability” OR “Heart Rate Interval” OR “RR variability” OR “cycle length variability” OR “heart period variability” OR “autonomic function” OR “vagal control”	Exercise OR “Physical Exertion” OR “Physical Fitness” OR “BMI” OR “Body Mass Index” OR “Body Constitution” OR “Stress” OR “Work Performance” OR endurance OR fitness OR strength OR “overuse injury” OR “stress injury” OR recovery OR fatigue OR readiness OR resilience OR overtraining	16
Embase	“Solider” /exp OR “rescue personnel” /exp OR “police” /exp OR “fire fighter” /exp “Military Personnel” OR “Emergency Responders” OR “Police” OR “firefighters” OR “Sheriff” OR “Patrol Officer” OR “Law Enforcement” OR “tactical athlete” OR “cadet” OR “FBI” OR “officer”	“HRV” OR “Heart Rate Variability” /exp OR “Heart Rate Interval” OR “RR variability” OR “cycle length variability” OR “heart period variability” OR “autonomic function” OR “vagal control”	“Exercise” /exp OR “body mass” /exp OR “body composition” /exp OR resilience OR readiness OR hardiness OR “fitness” /exp OR “stress” /exp OR “heat stress” /exp OR “job stress” /exp OR “muscle stress” /exp OR “musculoskeletal stress” /exp OR “mental stress” /exp OR “job performance” /exp OR “mental recovery” /exp OR “exercise recovery” /exp OR “fatigue” /exp OR “muscle fatigue” /exp OR “posttraumatic stress disorder” /exp OR endurance OR fitness OR strength OR fatigue OR readiness OR resilience OR overtraining	43
DTIC	“Military Personnel” OR “Emergency Responders” OR “Police” OR “firefighters” OR “Sheriff” OR “Patrol Officer” OR “Law Enforcement” OR “tactical athlete” OR “cadet” OR “agent” OR “recruit” OR “FBI” OR “officer”	“HRV” OR “Heart Rate Variability” OR “Heart Rate Interval” OR “RR variability” OR “cycle length variability” OR “heart period variability” OR “autonomic function” OR “vagal control”	Exercise” OR “Physical Exertion” OR “Physical Fitness” OR “BMI” OR “Body Mass Index” OR “Body Constitution” OR “Stress” OR “Work Performance” OR endurance OR fitness OR strength OR “overuse injury” OR “stress injury” OR recovery OR fatigue OR readiness OR resilience OR overtraining	7

### Article Screening

Review studies, conference proceedings and other literature that was not primary, not peer-reviewed, not available in English or had no available abstract was also rejected manually if not captured by search filters. Article titles clearly irrelevant to the topic (e.g., drug intervention studies, *in vitro* studies) were also removed. Articles reporting results from animal studies, a human population that did not include active or veteran tactical personnel, and studies using HRV as an intervention rather than a measurement (e.g., biofeedback device studies) were also screened out for the purposes of this review.

### Inclusion and Exclusion Criteria

Developed inclusion criteria were applied to the remaining titles: tactical personnel or trainees (well or unwell), veterans, measurement of HRV, assessment of a health/fitness/occupational outcome, use of HRV analysis findings to predict, measure or monitor a state of disease, physical or occupational performance. All study design types were considered, provided that HRV analyses were reported in relation to another factor. Article relevance to the question was first screened by CT. An additional author verified inclusions and exclusions (RO). Disagreements were adjudicated by a third author, BS.

### Quality Assessment

The Critical Appraisal Skills Programme (CASP) toolkit (Middle Way, Oxford, UK) (25) was selected for methodological assessment of the included studies. This assessment system has been used in previous systematic reviews and allows for fair and equitable assessment of a variety of study types (26). Three CASP checklists were ultimately necessary to evaluate the selected studies; the cohort study checklist, comprised of 13 total questions, the case-control checklist, comprised of 11 questions, and the randomized control trial checklist, also of comprised 11 questions. The randomized control trial checklist was the most suitable for quasi-experimental studies included in this review. The first two questions of each checklist screen for validity, and the following 9-11 questions guide reviewers through the assessment of study results, relevance of the results, methodology, and applicability. On the cohort study checklist, Question 7 simply asks for a summary of the results. This question was omitted, resulting in a final maximum score of 12. For each question that could be answered dichotomously, a publication was awarded with 1 point for answers of “yes” and 0 points for answers of “no” or “can't tell.” The case-control and randomized-control trial checklists required no modification beyond score quantification. For any questions which were not answered dichotomously within the checklist, an objective parameter was fit to the question. For example, on all checklists, there is a question pertaining to the precision of the results, phrased, “how precise were the results?” This question was awarded a “1” (yes) if exact measurements of significance, rather than inequalities, were reported. For other questions asking for treatment effect sizes, publications were awarded “1” point (yes) for including power or effect size analysis, and “0” points (no) if no determination was made as to the effect size. Disagreements in CASP scoring between authors were resolved by consensus. A referee (RO) was appointed prior to initiation of scoring. Screening and quality assessment were completed 2 February 2020.

## Results

The results of the search, screening, and selection processes are summarized in the Preferred Reporting Items for Systematic Review and Meta-Analyses (PRISMA) flow diagram (Figure 1). A total of 296 citations were captured in the finalized search, executed on 9 October 2019. At that time, all eligible results were downloaded, retained and screened in accordance with the screening methodology. A total of 56 duplicates were removed, leaving 233 titles. Of these, 194 were identified as irrelevant to the research question. From the remaining studies, the following exclusion criteria were developed: post-traumatic stress disorder (PTSD) or depression were the condition of interest (*n* = 10), an environmental condition was a key variable in the study (*n* = 2), HRV was measured, but not linked to a health and fitness or occupational outcome (*n* = 7). The remaining 20 publications were retained for this review. Further screening for quantitative review was considered, but ultimately not possible due to the heterogeneity of the included studies. The review was completed on 14 March 2020.

### Critical Appraisal

The mean CASP of the quasi-experimental studies was 9.67 ± 0.58 of 11 maximum points. The mean CASP of the case-control studies was 10.5 ± 0.7 of 11 maximum points, and the mean CASP of the cohort studies was 10.73 ± 0.78 of 12 maximum points.

### Data Extraction

Details of all relevant data retrieved from each included study can be found in Table 2. The Author(s), title, year, participants, demographics as available, anthropometrics as available, the performance metric(s) and key results, significant or not, as well as the final CASP score are included. The HRV analysis or analyses selected and their results are also included if succinct summarizations of the data were possible. Studies are organized first by design methodology, then alphabetically.

**Table 2:** Data extraction and summary of selected studies.

**References/Title**	**Participants**	**HRV analysis**	**Performance measurement**	**Results**	**CASP Score**
Gamble et al. (27) Different profiles of decision making and physiology under varying levels of stress in trained military personnel	Male US infantry soldiers/SRT operators (*n* = 26) Age: 30.73 ± 7.71 yrs	Low Frequency domain (LF) High Frequency domain (HF) LF/HF Ratio	Threat identification simulation: accuracy, sensitivity Low stress and high stress shoot conditions	Basal HF-Accuracy: positive relationship, *p* = 0.0379 Basal HF-sensitivity: positive relationship, *p* = 0.0379 LF/HF-stress condition: negative relationship, *p* = 0.0379	10/11 Quasi-Experimental
Marcel-Millet et al. (28) Physiological responses and parasympathetic reactivation in rescue interventions: The effect of the breathing apparatus	French Firefighters (*n* = 28 males) Age: 37 ± 7 yrs Height: 179 ± 6 cm Weight: 76 ± 9 kg BMI: 24 ± 2 (*n* = 6 females) Age: 29 ± 3 yrs Height: 171 ± 4 cm Weight: 64 ± 3 kg BMI: 22 ± 1	SDNN LnRMSSD	Three load conditions: personal protective clothing only (PPC); PPC and the full self-contained breathing apparatus (SCBA); and with PPC and only the cylinder of the SCBA (SCBAc) One baseline unloaded condition: Intermittent Fitness Test (IFT) VO_2max_: 54.3 ± 4.9 ml/kg/min	PPC-SCBA, SDNN: 27.8 ± 14.1-21.4 ± 9.2 (*p* <0.05) IFT-PPC, LnRMSSD: 2.4 ± 0.5-2.1 ± 0.5 ms *p* <0.01 IFT-SCBAc, LnRMSSD: 2.4 ± 0.5-2.0 ± 0.5 *p* <0.01 IFT-SCBA, Ln RMSSD: 2.4 ± 0.5-2.0 ± 0.5 *p* <0.01	10/11 Quasi-Experimental
Sanchez-Molina et al. (29) Effect of Parachute Jump in the Psychophysiological Response of Soldiers in Urban Combat	Male Spanish Army Soldiers (*n* = 19) Age: 31.9 ± 6.2 yrs Height: 173.6 ± 5.3 cm Weight: 73.8 ± 8.3 kg BMI: 24.2 ± 2.3 Experience: 12.8 ± 7 yrs	RMSSD LF HF ΔHF power	Simulated Parachute infiltration or ground infiltration into urban combat simulation Blood lactate Blood oxygen saturation (BOS) Rate of perceived exertion (RPE)	Blood Lactate increased significantly at the end of the simulation regardless of infiltration method (*p* <0.05) RMSSD and HF domain decreased while LF increased regardless of infiltration method (*p* <0.05) RMSSD-ΔLactate: *r* = −0.504 (*p* = 0.039) LFpost-ΔLactate: 0.589(*p* = 0.013) HFpost-ΔLactate: *r* = −0.589 (*p* = 0.013) ΔHF-BOS: *r* = 0.493 (*p* = 0.044)	9/11 Quasi-Experimental
Porto et al. (30) Firefighters' basal cardiac autonomic function and its associations with cardiorespiratory fitness	Two groups of Male Brazilian Firefighters (*n* = 38, on-duty) Age: 41 ± 11 yrs BMI: 26.1 ± 7.8 (*n* = 26, off-duty) Age: 40 ± 12 yrs BMI: 26 ± 13	pNN50: CRF <12METs: 1.3 CRF>12METs: 3.6 RMSSD: CRF <12METs: 18.3 CRF>12METs 23.8 LF/HF ratio: CRF <12METs: 5.2 CRF>12METs: 3.3	Maximal metabolic equivalent capacity (METs): VO_2max_ (on-duty): 42.4 VO_2max_ (off-duty): 40.0 Groups divided into <12METs or >12METs On duty status	Significant differences between those with METs <12 and >12: PNN50 (*p* = 0.07) RMSSD (*p* = 0.03) LF/HF Ratio (*p* = 0.01) No difference between duty status	11/11 Case-Control
Sanchez-Molina et al. (31) Assessment of Psychophysiological Response and Specific Fine Motor Skills in Combat Units	Two groups of male Spanish Army Soldiers (*n* = 19 Light Infantry) Age: 30.2 ± 5.25 yrs Height: 176.15 ± 8.31 cm Weight: 77.93 ± 10.25 kg	RMSSD LF HF	Simulated Urban Combat Competitive State Anxiety Inventory (CSAI-2R)	RMSSD, LF and HF values were significantly different between the light and heavy infantry groups (*p* <0.001): RMSSDpre: Light: 38.26 ± 40.69 Heavy: 164.89 ± 73.16 LFpre:	10/11 Case-Control
	BMI: 25.00 ± 3.11 Experience: 9.95 ± 5.17 yrs (*n* = 12 Heavy Infantry) Age: 34.5 ± 4.85 yrs Height: 177.42 ± 7.28 cm Weight: 79.21 ± 9.57 kg BMI: 25.10 ± 1.8 Experience: 14.58 ± 4.87 yrs			Light:76.60 ± 10.75 Heavy: 19.25 ± 13.11 HFpre: Light: 35.10 ± 25.51 Heavy: 80.59 ± 13.41 RMSSDpost: Light: 7.62 ± 5.62 Heavy: 88.68 ± 4.45 CSAI-2R correlated with all HRV measures: HFpre: *r* = 0.382(*p* = 0.034) LFpre: *r* = 0.406 (*p* = 0.24) RMSSDpre: *r* = 0.416 (*p* = 0.022) HFpost: *r* = −0.487 (*p* = 0.006) LFpost: *r* = 0.424(*p* = 0.020) RMSSDpost: *r* = 0.433 (*p* = 0.017)	
Andrew et al. (32) Adiposity, muscle and physical activity: Predictors of perturbations in hear rate variability	Buffalo, NY (USA) Police Officers (*n* = 360) Age: 42.1 ± 7.66 yrs BMI: 29.0 ± 4.40 Resting HR: 64.0 ± 8.82 bpm	Low Frequency domain natural log (LnLF): 5.31 ± 0.91 High frequency domain natural log (LnHF): 4.96 ± 1.13	Physical Activity Recall Questionnaire (Ln Physical Activity Index): 2.66 ± 1.04	Significant (*p* <0.05) correlation between LnPA and HRV: LnLF: *r* = 0.071 LnHF: *r* = −0.035	11/12 Cohort
Andrew et al. (33) Police work stressors and cardiac vagal control	Buffalo, NY (USA) Police Officers (*n* = 259 males) Age: 41.2 ± 6.8 yrs (*n* = 87 females) Age: 40.7 ± 5.5 yrs	Low Frequency domain natural log (LnLF) High frequency domain natural log (LnHF)	Spielberger Police Stress Survey	No significant correlations for men Significant (*p* = 0.024) inverse relationship between the “lack of support” stressor and LnHF for females	10/12 Cohort
Delgado-Moreno et al. (34) Combat Stress Decreases Memory of Warfighters in Action	Male Spanish Army Soldiers (*n* = 20) Age: 35.4 ± 6.2 yrs Height: 179.9 ± 7.0 cm Weight: 82.38 ± 10.5 kg BMI: 25.7 ± 2.6)	Low Frequency domain (LF), Pre: 48.3 ± 4.2, Post: 74.8 ± 14.4 High Frequency domain (HF), Pre: 51.7 ± 3.1 Post: 25.1 ± 14.3 All values in normalized units (n.u.)	Urban Combat simulation Post-mission event recall questionnaire Body Temperature, Pre: 37.8 ± 1.2°C, Post: 37.5 ± 1.3°C	Significant correlations between the following: LFpost, temp post: *p* = 0.035, *r* = 0.473 LFpost, correct sound recall: *p* = 0.020, *r* = −0.516 HFpost, temp post: *p* = 0.035, *r* = −0.474 HFpost, correct sound recall: *p* = 0.019, *r* = 0.517	10/12 Cohort
Diaz-Manzano et al. (35) Higher Use of Techniques Studied and performance in melee combat produce a higher psychophysiological stress response	Male Spanish Army Soldiers (*n* = 19) Age: 28.8 ± 4.9 yrs Height: 176.2 ± 5.3 cm Weight: 75.1 ± 5.3 kg BMI: 24.2 ± 1.3	RMSSD pNN50 HF (nu) LF (nu) HF/LF Ratio SD1 SD2	Hand-to-hand combat training drill Analyses divided by higher performing half vs. lower performing half (HPG, LPG)	Significant pre-post differences for all HRV measures in the HPG Significant differences for all HRV measures in the LPG, *except* HF (*p* = 0.880), LF (*p* = 0.164) and HF/LF ratio (*p* = -.140)	10/12 Cohort
Duarte et al. (36) Efforts of Patrol Operation on Hydration Status and Autonomic Modulation of Heart Rate of Brazilian Peacekeepers in Haiti	Male Brazilian Army Soldiers (n =20) Age: 23.5 ± 4.7 yrs Height: 175.1 ± 6.8 cm Weight: 74.6 ± 7.9 kg	AVNN, Pre: 1009.1 ± 119.3 ms Post: 862.7 ± 136.7 ms Low Frequency domain (LF) (nu) Pre: 50.5 ± 17.1 Post: 60.6 ± 13.9 High Frequency domain (HF) (nu) Pre: 49.5 ± 16.9 Post: 39.3 ± 13.8 LF/HF Ratio Pre: 1.49 ± 1.47 Post: 2.12 ± 1.19	Estimated Energy Expenditure (EE) Estimated VO_2max_ (Cooper's Test) Estimated Hydration Status (%Body Mass Change) Pre: 74.6 ± 7.9, Post: 72.9 ± 7.6	EE-LF/HF ratio: *r* = 0.49 *p* = 0.02 VO_2max_-LF/HF ratio: *r* = −0.42 *p* = 0.04 %Body Mass Change: *r* = 0.53 *p* = 0.02	12/12 Cohort
Grant et al. (37) The difference Between Exercise-Induced Autonomic Fitness Changes Measured After 12 and 20 weeks of medium to high intensity military training	South African Defense Force Recruits (*n* = 89 males) (*n* = 65 females) Age: 20.91 ± 1.29 yrs BMI: 22.85 ± 2.78	AVNN SDNN RMSSD pNN50 LF (nu) HF (nu) LF/HF ratio SD1 SD2	Basic Military training over 12 and 20 weeks VO_2max_: Basal: 49.54 ± 8.79 12 weeks: 54.14 ± 7.09 20 weeks: 54.15 ± 7.16	Significant differences in Mean RRI (*p* = 0.007), SDNN (*p* = 0.024), RMSSD (*p* <0.001) and SD1 (*p* <0.001) between weeks 12 and 20, but no changes in LF, HF or LF/HF Ratio	10/12 Cohort
Johnsen et al. (38) Heart Rate Variability and cortisol responses during attentional and working memory tasks in naval cadets	Royal Norwegian Naval Academy Cadets (*n* = 49) Age: 23.3 ± 8 yrs	RMSSD	California Computerized Assessment Package (Calcap) Computerized two-back test Background search task Cortisol level	Basal RMSSD-cortisol: *r* = −0.35, *p* <0.04 Basal RMSSD-post calcap: *p* <0.04 Basal RMSSD-post pop-out attention: *p* <0.04 Basal RMSSD-recovery: *p* <0.04	10/12 Cohort
Jouanin et al. (39) Analysis of heart rate variability after a ranger training course	Male French Military Academy Students (*n* = 23) Age: 21.7 ± 0.2 yrs Height: 176.5 ± 1.1 cm Weight: 74.0 ± 1.3 kg	AVNN Pre: 1008.4 ± 33.1 Post: 1177.5 ± 33.8 SDRR: Pre: 763.1 ± 18.8 ms Post: 837.5 ± 20.5 ms LF (nu) HF (nu) LF/HF ratio Total Power (TP)	French Ranger Training Course	Pre-Post training: Significant change in Mean RRI (*p* <0.001), TP (*p* <0.01), LF, HF and LF/HF ratio, (*p* <0.05)	10/12 Cohort
Kaikkonen et al. (40) Physiological Load and Psychological Stress During a 24-h Work Shift Among Finnish Firefighters	Professional Finnish Firefighters (*n* = 21) Age: 38 ± 7 yrs Height: 178 ± 7 cm Weight: 25 ± 2 kg BMI: 25 ± 2	RMSSD (ms): 24 h shift: 42 ± 14 6-h rescue: 38 ± 16 6-h Ambulance: 45 ± 21	24-h shift 6-h firefighting shift 6-h ambulance shift	Significant Difference between shift conditions (*p* <0.01)	10/12 Cohort
Lyytikäinen et al. (41) Recovery of rescuers from a 24-h shift and its association with aerobic fitness	Professional Finnish Fire/Rescue personnel (*n* = 14) Age: 34 ± 9 yrs Height: 178 ± 7 cm Weight: 80.8 ± 11.4 kg	SDNN RMSSD VLF LF HF	24-h rescue shift VO_2max_: 51 ± 9 ml/kg/min	Significant difference in SDNN: On shift-2nd day: *p* <0.05 On shift-third day: *p* <0.001 Significant difference in LF/HF ratio:	10/12 Cohort
		LF/HF ratio Total Power		on shift-2nd day: *p* <0.05 On shift-third day: *p* <0.01 No significant effect of VO_2max_ on HRV during recovery	
Oliveira-Silva et al. (42) Physical Fitness and Dehydration Influences on the Cardiac Autonomic Control of Fighter Pilots	Male Brazilian Fighter Pilots (*n* = 11) Age: 33.2 ± 3.2 yrs Weight: 76.0 ± 8.5 kg Height: 175 ± 5 cm BMI: 24.8 ± 2.3	RMSSD: Pre: 24.2 ± 8.2 ms Post: 20.8 ± 10.7 ms SDNN: Pre: 67.0 ± 29.1 ms Post: 77.3 ± 27.5 ms SD1: 17.5 ± 5.6 ms 16.4 ± 7.8 ms SD2: 79.3 ± 18.3 ms 74.7 ± 28.1 ms Sample Entropy (SampEn): Pre: 0.94 ± 0.22 Post: 0.86 ± 0.28 Alpha1 (fractal scaling)	Flight training Hydration status (hematocrit) VO_2max_ (Montreal University Track Test): 46.4 ± 5.66 Bench Press: 68.7 ± 10.8 kg Pull-Down: 70.0 ± 8.36 kg Leg Press: 201.3 ± 42.49	Flight Training day-rest day: RMSSD (*p* = 0.036), SDNN (*p* = 0.001), SD1 (*p* = 0.031) Significant negative correlation between hematocrit and RMSSD, SD1: *r* = −0.61-−0.81, *p* = 0.044-0.002 Significant relationship between VO_2max_-SampEn: *r* = 0.777, *p* = 0.001	12/12 Cohort
Rodrigues et al. (43) Stress among on-duty firefighters: An ambulatory assessment	Professional Portuguese Firefighters (*n* = 15 males) (*n* = 2 females) Age: 29.35 ± 8.85 yrs Experience: 9.41 ± 7.3 yrs	AVNN: 792.64 ± 92.29 ms LF/HF ratio: 3.82 ± 1.76	Perceived Stress Scale (PSS) Work events Normative ECG values: AVNN: 930 ± 133 ms LF/HF Ratio: 3.33 ± 3.47	Significant differences between fire, pre-hospital assistance, and accidents: LF/HF ratio (*p* <0.01) Significant differences between firefighter and normative ECG values: Mean NNI (82% of firefighters lower) LF/HF ratio (71% of firefighters above)	11/12 Cohort
Rodrigues et al. (44) Wearable biomonitoring platform for the assessment of stress and its impact on cognitive performance of firefighters: An experimental study	Professional Portuguese Firefighters (*n* = 19 males) (*n* = 2 females) Age: 29.90 ± 8.83 yrs Experience: 8.33 ± 8.21 yrs	AVNN SDNN RMSSD pNN20 pNN50 LF/HF ratio	Trier Social Stress Test (TSST) 2-choice reaction time task (CRTT)	CRTT1-Mean NNI: significant decrease, *p* <0.005 TSST-Mean NNI: significant decrease, *p* <0.005 TSST-SDNN: significant increase, *p* <0.001 LF/HF ratio-CRRT2: significant increase, *p* <0.001 LF/HF ratio-TSST: significant increase, *p* <0.001	12/12 Cohort
Shin et al. (45) Factors related to heart rate variability among firefighters	Professional Male South Korean Firefighters (*n* = 645)	SDNN RMSSD LnLF LnHF LF/HF Ratio	Korean Occupational Stress Scale (yes or no answers to a variety of occupational variables)	Smoking-RMSSD Yes: 31.96 ± 16.92 No: 28.69 ± 15.31 (*p* <0.05) Smoking-LnLF Yes: 5.73 ± 0.91 No: 5.54 ± 0.98 (*p* <0.05) Shift work-RMSSD:	12/12 Cohort
				Yes: 30.17 ± 16.54 No: 25.78 ± 11.11 (*p* <0.05) Job Demand-RMSSD: Low: 30.96 ± 16.41 High: 28.04 ± 14.97 (*p* <0.05)	
Souza et al. (46) Resting vagal control and resilience as predictors of cardiovascular allostasis in peacekeepers	Male Brazilian Army Soldiers (*n* = 50) Age: 25.4 ± 5.99 yrs BMI: 23.2 ± 4.34	AVNN RMSSD 2 min RMSSD 5 min	Trier Social Stress Test Ergo Resiliency Scale (ER-89)	Significant Spearman's Correlations for the following: Basal Mean RRI-speech task: *r* = 0.50, *p* = 0.0003 Basal Mean RRI-arithmetic task: *r* = 0.54, *p* <0.0001 Basal Mean RRI-recovery (speech): *r* = 0.45, *p* = 0.001 Basal Mean RRI-recovery (arithmetic): *r* = 0.53, *p* <0.0001 ER-89, RMSSD: *r* = 0.28, *p* = 0.05	11/12 Cohort

### Research Metadata

#### Study Designs

Three study design methodologies were utilized in the selected studies. Quasi-experimental studies (27–29) and case-control studies (30, 31) represented a total of four included studies. All other studies were of a cohort design. The follow-up period for the cohort studies varied from single instances of data collection activity, such as administration of a questionnaire and a single ECG trace, to as long as 20 weeks of follow-up in one study (37).

#### Demographics

Selected publications included males only in 11 studies, both males and females in seven studies and the gender distribution was not reported in three studies. Seven tactical subpopulations were represented; general law enforcement officers (32, 33), Army (27, 29, 31, 34–36, 46), Defense Force trainees (37), Naval cadets (38), Army Ranger trainees (39) firefighters/rescue personnel (28, 30, 40, 41, 43, 45), and Air Force fighter pilots (42). Specifically, the Army personnel were members of either the US, Brazilian or Spanish militaries. For the Fire/Rescue personnel, members were serving in French, Brazilian, Finnish, Portuguese, or South Korean communities. A total of nine countries were represented: USA (27, 32, 33), Spain (29, 31, 34, 35), Brazil (30, 36, 42, 46), South Africa (37), Norway (38), France (28, 39), Finland (40, 41), Portugal (43, 44), and South Korea (45).

### HRV Analyses

Analytical methods vary widely in their mathematical approach and provide different information on different autonomic processes. Table 3 describes the HRV analysis methods discussed in this review and provides an expansion of each acronym, a brief explanation of the measurement, and its clinical implications.

**Table 3:** Expansion and definition of HRV measurement acronyms.

**Measurement**	**Units**	**Expansion**	**Application**	**Interpretation**
RMSSD	ms	Root-mean square of successive differences	Primary measurement for short-term vagally mediated changes (2)	Time-Domain Measurements generally increase with increased aerobic capacity (2, 47)
pNN20	%	Percentage of adjacent intervals that differ by more than 20 ms	Assessment of vagal activity (2) Thresholds of ~6.8% or lower for disease states have been posited (48, 49)	
pNN50	%	Percentage of adjacent intervals that differ by more than 50 ms		
AVNN	ms	Average value N-N; mean interbeat interval	Sensitive to changes in Psychological stress (50)	
SDNN (SDRR)	Ms	Standard deviation of all interbeat intervals during the sample period	Most widely acceptable HRV measurement for assessing cardiac risk (24-h recording only) (2)	
VLF	ms^2^	Very-Low Frequency Band power (0.0033-0.04 Hz)	Intrinsically generated by the heart (2) Modulated by sympathetic activity (2)	Low VLF power has been associated with cardiac death, PTSD and inflammation (2, 51)
LF	ms^2^	Low Frequency Band power (0.04-0.15 Hz)	Represents baroreceptor activity during resting measurements (2)	Can be used to estimate vagal tone (2, 52)
LnLF	nu	Natural log of LF	Relative frequency band measurement to total power (53)	
HF	ms^2^	High Frequency Band power (0.15-0.4 Hz)	Parasympathetic and respiratory activity (2)	Low HF values are associated with high stress (50) Correlated with RMSSD (2)
LnHF	nu	Natural log of HF	Relative frequency band measurement to total power (53)	
LF/HF	%	Ratio of LF to HF power	For resting seated measurements, likely measures PNS and baroreceptor activity (2)	Highly dependent on measurement conditions (2)
TP	ms^2^	Total Spectral power	Useful for determining relative power measurements (2)	Broad estimate of total autonomic activity (2)
SD1	ms	Standard Deviation from y-axis on Poincare plot	Nonlinear measurement, useful for short-term analysis without trend sensitivity (2)	Predicts RMSSD, pNN50, SDNN, and power in the LF and HF bands, and total power during 5 min recordings (2, 52)
SD2	ms	Standard Deviation from x-axis on Poincare plot	Nonlinear measurement, useful for long-term analysis without trend sensitivity (2)	Correlates with LF power (2)
SampEn	–	Sample entropy or approximate entropy	Quantification of the unpredictability of a time series (2)	Useful for short recordings which may have noise (2)
Alpha1	–	Short-term detrended fluctuation analysis Extracts the correlations between successive RR intervals over different time scales	Reflects baroreflex activity (2)	Useful when longer recordings are not available (2)

#### Frequency-Domain and Nonlinear Analyses

The low-frequency band (LF) and high frequency band (HF) spectral power were the most popular analytical methods, with 18 studies documenting these HRV characteristics. Other spectral analyses included LF/HF ratio (27, 30, 36, 37, 39, 41, 44, 45, 54), total power (TP) (39), and the very-low frequency band (VLF) (41). The nonlinear analyses included *alpha1* (α1), a measure of signal self-similarity (42), sample entropy (SampEn) (42), SD1, and SD2 (35, 37, 42).

#### Time-Domain Analyses

The root-mean square of successive differences (RMSSD) was the most popular time-domain analysis, with 11 studies documenting this HRV characteristic. Other time-domain analyses included pNN20 (54), pNN50 (30, 35, 37, 54), mean interbeat interval, or AVNN (36, 37, 39, 43, 46, 54), or SDNN/SDRR (28, 37, 39, 41, 42, 45, 54).

### Aerobic Fitness

A total of four studies examined the relationship between one or more HRV metrics and aerobic fitness (VO_2max_) (30, 36, 41, 42). Three of the four were cohort study designs (36, 41, 42), and one was quasi-experimental (30). Three (30, 42), including the experimental study (36), found significant relationships between one or more HRV values and aerobic fitness, and one did not (41). The study finding no significant relationship examined only the LF/HF ratio and SDNN HRV indices. Of the studies reporting significant relationships, LF/HF ratio (36, 41), sample entropy, PNN50, and RMSSD were examined. The pooled mean VO_2max_ across all four studies was 45.4 ± 6.2 ml/kg/min, with the lowest VO_2max_ in the cohort of off-duty male Brazilian firefighters (40.0 ml/kg/min) (30) and the highest in the cohort of male Brazilian peacekeepers (52.9 ml/kg/min) (36).

### Other Fitness Measurements

Two studies (32, 42) examined fitness variables that were not estimates of aerobic capacity. One recorded one-rep maximum strength measurements from Brazilian fighter pilots (bench press, pull-down and leg press) and found no significant relationships between HRV indices measured on a rest day or on a flight training day and those strength tests. The other study (32) found a significant correlation between self-reported physical activity levels and the LF and HF signal strengths.

### Tactical Scenario Simulations

Six studies utilized a simulated combat scenario for participant testing (27, 29, 31, 34, 35) or observed an actual tactical engagement (36). Of these studies, all found significant pre-event to post-event changes in HRV. Specifically, one study (35) comparing differences between high-performing and lower-performing soldiers in hand-to-hand combat training drills found significant time-domain differences (RMSSD, PNN50, SD1, SD2) between groups, but no differences in spectral-domain analyses. In the other studies, spectral analyses correlated with weapon accuracy and threat discrimination sensitivity (27), estimated energy expenditure, and hydration status as measured by percentage of body weight change of the course of the observation period (36). One study in this category compared a light infantry unit to a heavy unit in a simulated ground combat scenario (31). The light infantry unit, acclimated to such events and training, showed significantly different spectral responses when compared against the heavy infantry unit.

### Cognition Testing

Three studies included a measurement of cognitive performance (34, 38, 43). Of these three, one found significant correlations between sound recall and the LF domain following a combat simulation (see section above) (34). One found significant relationships between greater basal RMSSD and two computerized cognitive stress tests, as well as during recovery from those tasks (38). The last study found a significant decrease in AVNN at the start of a two-choice critical reaction time task and a significant change in LF/HF ratio following the task (43).

### Occupational Stress

Three studies (33, 45, 46) measured occupational stress directly through surveys or through a tactically relevant evaluation tool. Andrew et al. (55) found an inverse correlation between lnLF and the “lack of support” stressor; meaning that greater lack of support correlated with less vagal control as measured by LF power. Shin (45), in a sample of 645 professional South Korean firefighters, found significantly reduced RMSSD values in those that reported smoking, reported “high” vs. “low” job demand, and those that reported shift work. Significantly higher lnLF values were also reported in smokers. Souza et al. (46) measured trait resilience in a cohort of Brazilian Army soldiers using the ER-89 scale and found a correlation between trait resilience and RMSSD.

### First Response Shift Work

Three studies assessed cohorts of fire and rescue personnel working 24-h shifts (40, 41, 43). Kaikkonen et al. (40) found a significant difference in RMSSD between the 6-h firefighting and 6-h ambulance phases of professional Finnish firefighters' shifts, with the rescue phase of the shift resulting in a decrease of RMSSD. Lyytikäinen et al. (41), previously mentioned above, examined HRV changes during the recovery days following a 24-h shift. They found significant differences in the SDNN and LF/HF ratio from days 2 to 3 of recovery. Rodrigues et al. (44) found significant differences in LF/HF ratio during a variety of fire and rescue tasks. Specifically, the LF/HF ratio increased most during response to accidents. They also compared their mean N-N interval data to normative healthy adult data and found the rescue personnel lower AVNN values for rescuers, as well as higher LF/HF values.

### Training Events

Two studies (37, 39) followed participants through a period of tactical training. One (39) measured differences in AVNN, TP, LF, HF, and LF/HF ratio between the start and end of French Ranger training. They found significant changes in all measured values. Grant et al. (37) assessed the differences in aerobic capacity and HRV values at one 12 and 20 weeks during South African Defense Force initial entry training. They found that while the mean AVNN, SDNN, and RMSSD continued to improve between 12 and 20 weeks, the LF/HF ratio was not significantly different over the same time period (37).

## Discussion

The aim of this review was to identify primary studies examining relationships between HRV and relevant health, fitness and operational outcomes in tactical personnel, critically appraise the methodological quality of the identified studies, and summarize the results. The quality of the 20 included studies was generally high and included a variety of observational and quasi-experimental designs over a wide range of follow-up periods. While observational research was necessary for the aims of most studies, and variations in study designs and research questions covered a variety of topic in which single instance recordings were appropriate, few of the studies included in this review were longitudinal, and larger, more comprehensive datasets describing the HRV characteristics of tactical personnel in a variety occupational fields and settings would be beneficial. Further, the CASP checklists do not account for sample sizes, which were generally small, with a few notable exceptions, so while the methodology of the included studies may have been sound, caution must be taken when interpreting the results. Qualitatively, however, the studies included in this review generally agreed, and did indicate that HRV is an effective tool for measuring psychophysiological stress across professional environments and often correlates effectively with a wide variety of outcomes of interest. The included studies indicate that HRV measurements can be effectively applied across a wide variety of tactical settings.

### Physical Fitness

Of the four studies investigating the relationships between HRV measures and aerobic fitness, only one found no significant association, and the mean VO_2max_ of 51 ml/kg/min was higher than all but one of the other studies. This could suggest a ceiling effect, indicating that aerobic fitness above a certain capacity within a certain profession lends no additional benefits to recovery, but all considered studies used an indirect, estimated method to determine VO_2max_. Additionally, a variety of HRV analysis methods were used, and while frequency domain values did not trend significantly with physical fitness in all studies, the study by Oliveira-Silva et al. examining the cohort with the highest aerobic capacity did find a significant relationship when examining sample entropy (42), suggesting that non-linear methods may be the most suitable method for these study designs.

Further research in this area might focus on directly measuring aerobic capacity of the cohort, and conducting similar research investigating VO_2max_, HRV and recovery in different tactical professionals. Larger sample sizes may also clarify these emerging relationships. So far, only one study has examined any measurements of strength, and no measures of power have been linked to HRV indices. While no significant associations were found in a cohort of fighter pilots, it may be possible that fitness within the strength and power domains contributes to individual perception and response to stress, as well as recovery from stressful exposures in other tactical settings.

### Tactical Scenarios

All six studies measuring HRV during simulated or actual combat situations found significant changes. While not unexpected, these results nonetheless provide a foundation on which organizations can begin to consider implementing additional psychophysiological monitoring of personnel to assist in training and deployment decisions. While these studies achieved high levels of external validity, by exposing personnel to rigorously designed and realistic simulations, questions remain as to which factors within these scenarios most significantly affected the HRV indices of operators, and therefore, which components require further validation or incorporation into regular training. For example, it is possible that load carriage, a common task for tactical personnel, may influence chest biomechanics and perceived stress, independently altering HRV. Previous research has indeed demonstrated that even experienced tactical personnel report increased exertion when carrying a load, even if physiological measures, such as estimated energy expenditure, do not significantly change (56). Spectral and time domain analyses of HRV may be able to quantify and explain these anomalies. Furthermore, while one study has conducted a threat identification scenario, live firearms were not used, and further research into the specific effect of live firearms operation on HRV may be necessary, and may provide additional information on underlying mechanisms that contribute to the effectiveness of deliberate HRV modulation as an intervention for improving tactical decision making (57). For field measurements and analyses of HRV in these scenarios and similar scenarios, selecting more robust analyses, such as nonlinear and fractal-scaling methods may again be the most suitable.

### Cognition Testing

Making decisions under pressure and other cognitive work is critical to the success of many tactical operations, and a key skill for ensuring operator and public safety. While the studies examining cognitive stress and HRV values are limited to only three, which represented the military and firefighting professions, the results are promising. The value for organizations may come primarily from the ability of HRV to discriminate and quantify the severity of responses to cognitive demands without relying solely on subjective feedback. The results from one study in particular by Delgado-Moreno et al., which assessed recall capability and spectral HRV analysis (34), may demonstrate that personnel who adapt to stressful stimuli struggle less with higher-order tasks. This type of analysis and its integration into training, especially in law enforcement, may be beneficial for improving decision-making training and promoting improved public safety.

### Occupational Stress

The study by Shin et al. (45) was the largest cohort studied by a considerable margin, with a total of 645 participants. The Korean Occupational Stress Scale, while a subjective tool, is a widely utilized measurement of occupational stress and was used as the primary instrument (58). They found that the organization system and occupational climate were correlated with lnHF changes. Time-domain changes were also found in firefighters that reported high job stress. This agrees with another large cohort of manual laborers in Korea and suggests that organizational structures and employee support can have significant changes in the allostatic load of personnel (59). Although the study by Shin et al. did not account for physical activity outside the workplace, the study by Kang et al. did, and determined that social support was an independent risk factor for adverse HRV changes. Therefore, it cannot be assumed that improvements in physical conditioning will be able to fully counteract the potential negative effects of job stress induced by organizational, job support or occupational climate concerns. As such, tactical organizations may benefit from monitoring not only external occupational stressors and hazards but may potentially benefit from assessing the effects of internal organizational and support impacts. Further research accounting for physical conditioning in tactical personnel, and enhanced qualitative methods assessing occupational organization, support, and climate may clarify some of the unique causal relationships between organization-induced occupational stress and allostatic load in tactical operations.

Likewise, in the study by Andrew considering police work stressors and cardiac autonomic balance, a significant association between a lack of organizational support and frequency-domain changes in HRV was found, but for female officers only (33). The authors concluded that chronic insufficient organizational support may lead to a loss of the cardioprotective effect females typically experience as a result of greater vagal control compared to men. Their results were consistent with another study that found relationships between organizational occupational stress and metabolic disorder in women, but not men (60). Given that females will typically form a minority within a tactical organization, specific regard to their occupational support may be especially necessary to support their health and effectiveness. One solution Andrew and his colleagues offer is for police organizations to provide for support coping opportunities, a method of stress coping more typically adopted by females. Further research may investigate the effect of occupational organization, support methods and climate with specific regard to female operators or trainees to determine optimal strategies for maximizing their performance.

### Shift Work

While shift work is endemic to many tactical organizations, the studies in this review represented exclusively fire and rescue personnel, and the results indicated that the autonomic impacts of shift work in tactical personnel may manifest differently than in other populations. For example, two separate studies in two very different geographical locations (Finland and Portugal), both found that fire and rescue personnel who were assigned to rescue duties, which included motor vehicle accident response, experienced significantly greater HRV changes than when they worked regular fire suppression duties (40, 43). Research in healthcare workers found limited differences between physicians and nurses who worked rotating shifts and those who worked day shifts only (61), indicating that research considering longer-term HRV measurements, such as the VLF component of the frequency-domain and the nonlinear SD2, may require careful interpretation in tactical settings.

Other work in this area to aimed at using HRV indices to measure physiological strain, which may then be used when determining work/rest cycles and other scheduling concerns. Time-domain analyses were the most common across these studies, but spectral analyses were also represented and were significant. While no significant associations were found between VO_2max_ and shift recovery (discussed above), it should be noted that the autonomic effects of shift work lasted up to three days (41). Given that males may be more susceptible to adverse HRV changes induced by circadian rhythm disruptions (62), and that males comprise the majority of most tactical organizations, the effects of shift work on autonomic regulation should be investigated further in a wide variety of tactical operations to develop stronger evidence to guide policy decisions. Further studies or reanalysis efforts may also aim to apply novel indices, such as sample entropy or other signal-self similarity assessments to strengthen the relationships between HRV measurement, physiological strain and operator deployment decisions. Given the differences in HRV responses to different duties even within a single unit, other populations that rely on rotating shifts, such as law enforcement and military organizations should also be considered separately to determine the unique influences present between professions and how to proceed with long-term HRV measurement interpretations.

### Training Events

Two studies followed trainees through a period of accession training, one a Ranger training course in France, and the other basic military training in South Africa. Both found that tactical training results in HRV changes across both time and spectral domains, and one was specifically using HRV to assess for the presence of overtraining syndrome (OTS) between the 12th and 20th week of training (37), as OTS is known to contribute to injury risk (63). A link between HRV and chronic musculoskeletal injury has been proposed in endurance athletes (13), and if proven effective, tactical organizations may be able to screen and triage trainees before clinical symptoms develop, reducing the burden of injury during training. One recent study in Crossfit™ athletes found significant associations when investigating the relationships between workload and HRV in the time domain (64). Given the similarities between Crossfit™ and tactical conditioning activities (14), this association may prove especially valuable for tactical training programs and warrants further investigation in larger cohorts of tactical personnel. As previously stated, more robust HRV analytical methods may also be beneficial for inclusion in further studies.

## Conclusions

The measurement and application of HRV indices to monitor psychological, physiological and more specifically, autonomic stress, encountered by the tactical operator continues to be developed and has made significant strides over the past decade in terms of utilization by a wide range of organizations, comparison to normative populations and quantification of the stress of tactical work. While strong evidence is still emerging, based on the quality and general agreement of studies included in this review, HRV monitoring appears to provide valuable insight into the psychophysiological responses of tactical personnel during occupationally relevant activities and recovery from those activities. However, substantial further research is still necessary. Specifically, the recruitment of larger cohorts and the collection of normative data specific to healthy tactical personnel should enhance result interpretation. Additionally, further isolation and analysis of specific variables relevant to the end user, such as the independent effects of load carriage, may help determine causal relationships, strengthen the reliability of applications and ultimately provide personnel with an additional tool to maximize health and performance. Finally, HRV may prove to be highly effective for mitigation of chronic musculoskeletal injury in tactical operators and trainees as a screening tool, but such associations require further exploration, in terms of both cohort selection and recruitment and HRV analysis method(s).

## Data Availability Statement

The raw data supporting the conclusions of this article will be made available by the authors, without undue reservation.

## Author Contributions

CT and RO: conceptualization, methodology, and writing—original draft preparation. CT: software, formal analysis, investigation, writing—review and editing, and supervision. RO and BS: resources, data curation, visualization, project administration, and validation. All authors have read, reviewed, and approved the current manuscript for submission.

## Conflict of Interest

The authors declare that the research was conducted in the absence of any commercial or financial relationships that could be construed as a potential conflict of interest.
